# Relationship between the Duration of Urban Nature and a Lower Waist-Hip Ratio

**DOI:** 10.3390/ijerph191811606

**Published:** 2022-09-15

**Authors:** Pongsakorn Suppakittpaisarn, Nadchawan Charoenlertthanakit, Ekachai Yaipimol, Vipavee Surinseng, Chulalux Wanitchayapaisit, Gunwoo Kim

**Affiliations:** 1 Landscape Design and Environmental Management Studio, Faculty of Agriculture, Chiang Mai University, Mueang Chiang Mai, Chiang Mai 50200, Thailand; 2 Department of Biology, Faculty of Sciences, Chiang Mai University, Mueang Chiang Mai, Chiang Mai 50200, Thailand; 3 Department of Environmental Engineering, Faculty of Engineering, Chiang Mai University, Mueang Chiang Mai, Chiang Mai 50200, Thailand; 4 Graduate School of Urban Studies, Hanyang University, 222 Wangsimni-ro, Seongdong-gu, Seoul 04763, Korea

**Keywords:** green infrastructure, obesity, adiposity, Southeast Asia, nature and human health

## Abstract

Obesity is a prevalent health issue. Evidence suggests that the availability of urban nature may reduce the risks of obesity. However, several knowledge gaps remain. This study explores the relationships between the dose (distance, duration and frequency) of urban nature and demographic factors towards obesity risks among people in Thailand. A total of 111 participants in three urban and peri-urban nature locations answered a survey regarding their distance from green spaces, frequency of visits, and duration of their typical stay, as well as their socio-demographics, and waist-hip ratio (WHR). The results suggested that at least 1–2 h per typical visit to nature predicted low-risk WHR in women. Male participants are more likely to have a high-risk WHR. Increasing age predicted low-risk WHR. Spending more time in green spaces predicted lower odds of high-risk WHR, while distance did not predict the odds. This research is one of the first to study the relationship between time spent in nature and obesity, and one of the first nature and health studies conducted in Thailand. Given that Thailand is one of the countries most affected by obesity in Southeast Asia, this study is relevant and essential. Future research should explore the quality factors of the park with longer duration of stay.

## 1. Introduction

Obesity has become a prevalent health issue worldwide [1,2,3]. Empirical evidence shows that urban nature may help reduce obesity risks [4,5,6], suggesting that designers, planners, and decision makers should provide accessible urban nature as an important part of the urban environment. However, critical questions remain about the relationship between urban nature and obesity. Additionally, there is little information about how a different dose of nature, including duration, frequency, density, and distance, may influence this relationship, or the extent to which the benefits of nature may differ among individuals depending on their ethnicity, sex, and age [7]. Knowledge on the extent to which these factors may affect the relationship between nature and human health, especially obesity, can enable practitioners such as designers, planners, and decision makers to design an evidence-based built environment that can have a positive impact on health. 

In this study, we examine questions regarding the dose of nature and demographic factors related to the risk of obesity among participants by visiting three urban and peri-urban nature locations in Chiang Mai, Thailand. 

### 1.1. Obesity and Human Health

Since 1975, the obesity rate has tripled worldwide. By 2016, 650 million adults were obese, with a Body Mass Index (BMI) over 30. This number accounted for 13% of the world’s adult population [8]. Obesity has been recognized as a serious health risk. An analysis of data between 1980 and 2013 by the Global Burden of Disease suggested that obesity is a growing health concern. However, despite this being the case, no successful studies regarding the prevention of obesity were reported in that 33-year period [9].

The problem of obesity stretches beyond rich and developed countries. Several studies have reported rapidly rising concerns about the obesity rate in developing nations [10,11,12], especially among the Pacific Islands, African nations, and the Middle East [10]. Meanwhile, the prevalence of obesity in the West Pacific region is lower than that of other regions; however, the rate in this region has also been increasing rapidly. For example, among the Chinese population, the obesity rate increased from 0.8% in 1980 to 4.9% in 2015 and from 1.7% in 1980 to 6.2% in 2015 in the Southeast Asian population [2]. This rise is alarming because Asian populations tend to have a higher body fat percentage compared to their European counterparts, indicating higher health risks for identical BMI [12].

The increasing obesity rate highlights concerns about the health of human beings. Overweight and obesity are the fifth leading causes of death worldwide, causing approximately 3.4 million deaths per year [13]. People living with obesity risk having many co-morbidities such as glucose intolerance, Type II diabetes, hypertension, fatty liver disease, musculoskeletal disorders, degenerative joint disease, and cancer [8,14]. In particular, they are at a higher risk of cardiovascular diseases, which was the leading cause of death in 2019 [15]. Treatment of obesity-related diseases also involves considerable healthcare costs. In the United States, 73 billion US Dollars was spent on obesity-related medical issues in 2009 [16] and a recent study reported that 190 billion US Dollars was spent on obesity and obesity-related medical issues, accounting for 21% of the healthcare expenditure in the United States [13]. 

These issues worsened during the recent coronavirus 2019 (COVID-19) pandemic. Most people spent their time indoors and became relatively sedentary [17,18]. Outdoor exercise during the pandemic were reported to have positive benefits that may outweigh the risk of infection [19]. However, people’s access to such places are limited, owing to the changes in public transportation, especially in developing countries [20]. Furthermore, lifestyle changes and the fear associated with the pandemic can cause chronic stress, which increases the risks of obesity [21]. In addition, COVID-19 also tends to have adverse and relatively lethal effects on people with obesity and obesity-related issues, such as sleep apnea [3,22]. 

### 1.2. Urban Nature and Obesity

The risk of obesity can be reduced through access to urban nature. Urban nature refers to the natural elements that exist in urban areas, such as street trees, neighborhood parks and open spaces, riverfronts, beaches, and nature trails. Systematic reviews have suggested that living closer to nature and having access to more blue and green spaces are associated with lower obesity rates in adults and older adults [6,8,9]. Recent observational studies conducted in Europe [4], Asia [23], and North America [24] found links between the availability of urban nature and the rates of obesity and adiposity.

Few mechanisms can explain the relationship between obesity and urban nature. For example, urban nature is a place for physical activities, which contribute to reduced obesity risks [6]. Previous studies about urban nature and obesity have agreed that physical activities form a part of the explanatory or contributing factors alleviating obesity [6,8,24,25], and some studies have also shown associations between green spaces and active recreation [24,26,27]. Furthermore, urban nature can include the walkability of a place, encouraging active daily lifestyle [28,29]. However, the quality of these green spaces, including their types and perceptions toward them, can influence people’s inclination to use such spaces [25,30,31,32]. 

Another underlying mechanism between urban nature and obesity may include chronic stress. Chronic stress can attribute to the storage of visceral fat, which is stored inside the stomach and is harmful to internal organs [21,33]. Furthermore, people who are stressed are likely to crave and consume more calorie-rich food than those who are not [34]. Therefore, stress regulation is important in obesity control. According to psycho-evolutionary theory, urban nature can reduce stress [35], thus reducing the risk of obesity. A rapidly growing body of literature posits that contacting nature in daily life helps reduce the levels of acute stress [36,37,38], and people with access to nature are less likely to have illnesses related to chronic stress [7,9]. Thus, we suggest that environmental designers, planners, and decision makers make urban nature accessible to everyone to provide them with health benefits, including reducing the risks of obesity and obesity-related health issues.

### 1.3. Critical Knowledge Gap: Doses and Demographics

While we understand the role of urban nature in reducing obesity risks, critical knowledge gaps remain. First, we are unaware of the extent to which the amount of urban nature, or dose of nature, may play a role in such a relationship [7,34,37]. Dose of nature includes how much nature we have around us (such as accessibility and density of nature), how often we spend time in nature (frequency), and how long we spend time in nature (duration) [34]. Among these doses, duration of nature is rarely explored. In other words, how much time should we spend in nature to gain optimal benefits? While some research has found that spending *one to* five minutes *with nature* can provide the highest mental health benefits [39,40], others have indicated that 10 min was sufficient to restore attention and help people recover from stress [36,38]. Another study found that in a range of 0–80 min, 20–30 min of nature exposure provided the best benefits [41]. Additionally, a typical American adult was recommended 150 min in total of moderate to vigorous physical activities throughout a week, but the number referred to any kind of exercises, rather than exposure to nature [42]. Overall, the collective results were inconclusive regarding the duration of urban nature exposure to gain optimal health benefits, and most studies have focused on mental health. For designers, it can be crucial to understand the duration to enable them to design and allocate spaces and experiences appropriate to that period. 

Another research gap includes the question of how our individuality impacts the way we gain benefits from urban nature [7]. In particular, to what extent do demographic factors, such as age, race, and sex, influence the relationship between urban nature and obesity? Sex plays a role in both urban nature benefits and obesity [43]. In a previous study, participants were stressed and asked to view nature in a different density; only men exhibited significant changes in stress recovery rate measured through cortisol, a biomarker for acute stress [38]. In another study, women and men’s perceptions of safety differed, predicting different use patterns [44]. In terms of obesity, adult men and women have different fat distribution and muscle mass production rates, leading to significant differences in obesity rate [43,45]. For example, in Thailand, adult women are more likely to be obese than men [46,47]. Furthermore, a person’s age plays an important role in their odds of obesity and how their body distributes fat [43,46]. According to a study of Finnish adults, means of waist circumference has increased by 2.7 cm in men and 4.3 cm in women over a duration of 15 years [48]. Another study from the United States found that the older population exhibit increasing BMI and waist circumference [49]. Sex and age have interactive tendencies in obesity and fat distribution. As people age, they are likely to store fat around the waist than their hips, impacting measurements of obesity [50]. Women’s reproductive stages, such as menopause, also affect obesity rates and tendencies [43]. 

Furthermore, socio-demographic factors, including race, occupation, culture, and geographical factors may affect adiposity and obesity. Upbringing, behaviors, and relationships with urban nature differ between cultures, and sometimes each region may develop unique needs for ecosystem services [51,52,53]. Furthermore, different races and cultural habits may affect adiposity. For example, Asian people tend to have higher fat percentage than their Western counterparts with the same weight, thus changing their BMI risks [12,54]. When studying a particular population group, other measurements of obesity, such as waist-hip ratio (WHR), are relatively suitable [46]. WHR identifies health risks by measuring participants’ waists and hips. If the value is higher than 0.9 for Asian men and 0.8 for Asian women, the person has higher health risks [55,56]. However, there is little evidence to demonstrate the relationships between urban nature and WHR. 

Recent discoveries suggested that while genetics plays a role in obesity, environmental factors also significantly contribute [57]. Thus, the differences in adiposity between socio-demographic factors may be due to several environmental factors, including structural socio-demographic oppressions [58]. For example, in the United States, racism and classism has limited some socio-demographic groups from accessing healthy food, nutrition and health education, and affordable healthcare; thus, people from a particular race and economic status are more likely to have higher adiposity [59,60]. Considering these factors, both cultures and oppressions can be combined into the Social Determinants of Health (SDoH) [61]. For Thailand, while most people were considered Southeast Asian in larger perspectives, smaller subraces exist. Aside from people from Central Thailand and Chinese-Thais, who are considered privileged in conventional Thai society, people from northern, southern, and northeastern Thailand have distinctive diets and cultures due to their diverse origins, and they face different, albeit mild, prejudices [62,63,64,65]. These prejudices are higher among Muslim Thais [66,67] and Hilltribes minorities [68]. Some occupations, such as physical laborers and home-makers, are also looked down upon in conventional Thai culture compared to other jobs due to lower wages [65]. Occupation may also play a role in their daily physical activities. These factors might contribute to similar limitations towards health and well-being, including adiposity, and the relationships between urban green spaces and adiposity. 

To explore how urban nature may benefit these population groups, studies must be carried out in these regions and measurements other than BMI, such as WHR, must be incorporated into studying the relationships between urban nature and obesity. 

Thus, this study aimed to answer the following research questions:

RQ.1. To what extent do the participants’ demographics and doses of urban nature (including distance, duration, and frequency) predict the WHR?
RQ.2. To what extent do the participants’ demographics and doses of urban nature (including distance, duration, and frequency) predict odds of high-risk WHR?

## 2. Methods

### 2.1. Study Site Selection 

**Figure 1:**
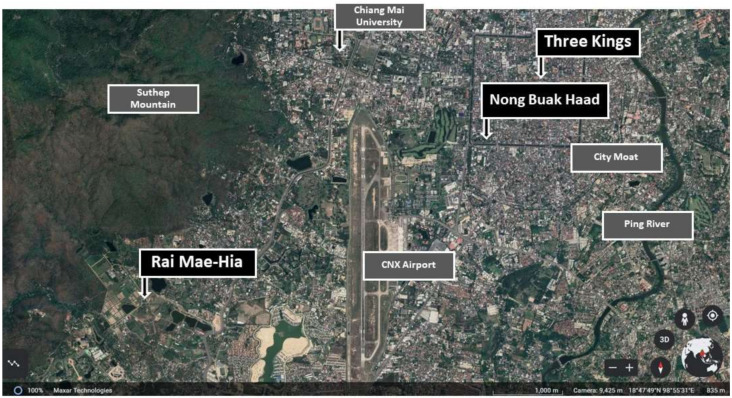
The location of the three study sites.

This study was conducted in Chiang Mai, Thailand. By 2017, Thailand had the second highest rate of overweight population in Southeast Asia [1]. Thus, the study was relevant to the region. Chiang Mai, a city situated in northern Thailand, is a popular tourist destination with many natural locations. It is adjacent to many national parks and suitable for natural activities such as birdwatching and hiking. However, the city lacks accessible urban green spaces [69]. Three urban and peri-urban sites were selected in Chiang Mai to represent different types of available types of urban nature: an urban farm (Rai Mae-Hia), an urban park (Nong Buak Haad Park), and an urban plaza (Three Kings Monument) (Figure 1). We received written permission from the related authorities to conduct our studies on these locations.

### 2.2. Participants and Recruitment

In this study, we recruited 111 adults aged 18 to 65 years old who visited one of these locations during the study period. All experimental and recruitment procedures were approved by the Research Ethics Committee of Chiang Mai University (protocol code 2564/074) on the 22nd of April 2021. The research team built on-site stations between June, July, and November 2021, leaving a gap during the monsoon season in Thailand, when it rains almost every day. The station was operational between 7 a.m.–9 a.m. and 4 p.m.–6 p.m., randomized within three places from Monday to Saturday. The team recruited the participants by presenting themselves as researchers from the Faculty of Agriculture, Chiang Mai University, and asked the participants whether they wanted to answer a five-minute survey.

### 2.3. Survey Questions

The survey was modified from Kim and Miller’s 2019 study [70], which investigated the behaviors, perceptions, and health of participants using Huckleberry Trail and the Heritage Community Park and Natural Area in Blacksburg, Virginia, thus it has previously been validated. The survey inquired about participants’ age groups, their biological sex, the distance between their residence and the green spaces they were in, the frequency of their visit, and the duration of their typical visit. For frequency, the participants were asked to choose one of five choices: first time, seldom, sometimes, often or regularly. For duration, the participants were asked to choose between one of three choices: less than 1 h, <1–2 h, and >2 h. For distance, the participants were asked to choose one of four choices: <1 km, 1–2 km, 2–5 km, and 5 km. 

For health information, the survey asked for measurements of their waist and hip circumference, their height, and their weight. We explained where the measurements may be obtained. If the participants were unaware of their measurements, we provided a measurement tape and instructed them on how to measure the circumference. 

We also collected demographic data including race (Central Thai, Northern Thai, Chinese-Thai, Muslim-Thai, and Other), living social condition (alone vs. with others), and occupation (physical job, desk job, homemaking, unemployed, students, and others). If participants identified with more than one, we asked them to choose what they identified with the closest. 

We conducted the study in accordance with the COVID-19 precautionary guidelines of the city. The researchers wore masks throughout the data collection period and provided hand sanitizers to the participants after they completed the survey. 

### 2.4. Statistical Analysis

To understand the relationships between distance, frequency, and duration of green infrastructure (GI) toward human WHR, three calculations were conducted. First, we calculated the BMI and WHR from their self-reported bodyweight, height, waist, and hip measurements. After that, we investigated the extent to which BMI and WHR of the participants were correlated with each other via Pearson’s Correlation Test. We then conducted Welch’s ANOVAs to understand whether different levels of doses (distance, duration, and frequency) of nature and age are associated with a lower WHR. The tests were conducted separately among male and female participants owing to differences in WHR requirements. The third test, Binary Logistic Regression, was conducted to understand whether these dose factors, along with the participants’ sex, influence the odds of having a WHR related to lower health risks based on the WHO standard: 0.9 for men and 0.8 for women [43]. 

## 3. Results

A total of 111 participants answered the survey, which represents the population size at a 90% confidence level with 8% margin of error. This number of participants are sufficient according to previous similar studies [70,71,72,73]. The demographics of the participants are presented in Table 1. Overall, the mean WHR was 0.9 (Standard deviation [SD] = 0.1) in men and 0.8 (SD = 0.1) in women, indicating that the average male and female participant had low-risk body compositions, which would not remain such if these values continued to increase. Additionally, participants had a mean BMI of 22.6 (S.D. = 3.8), which is considered a low-risk range. The statistics of their BMI and WHR are further explained in Table 2.

**Figure 2:**
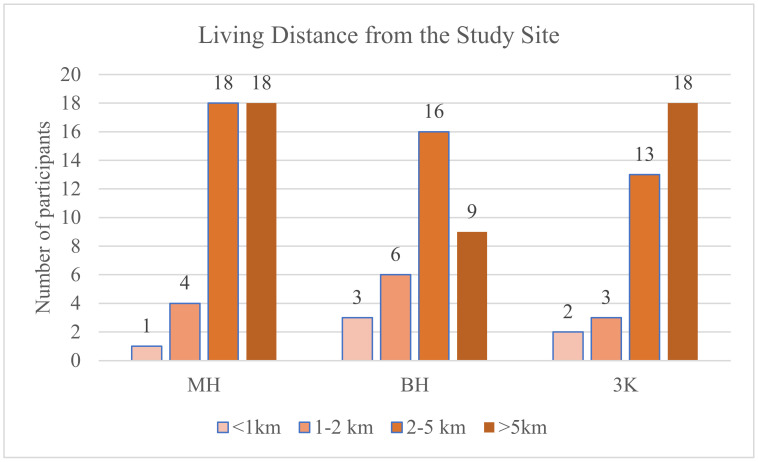

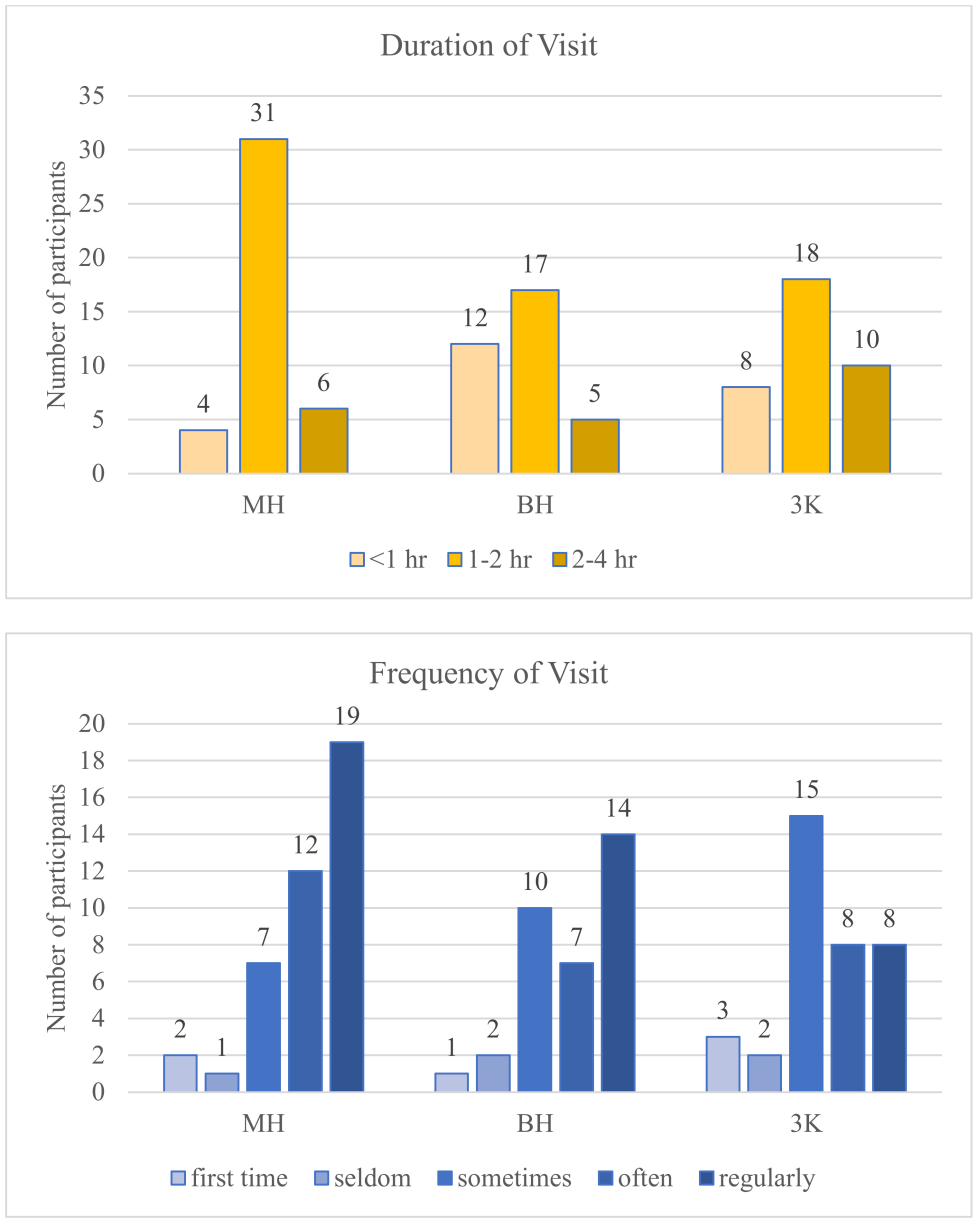
The frequencies of answers for distance (**top**), duration (**middle**), and frequency (**bottom**) across study locations. MH, Rai Mae Hia; BH, Buak Haad Park; 3K, Three Kings Monument.

Figure 2 illustrates the statistics regarding the estimated distance from the park, their visiting frequency, and the duration of a typical visit in green spaces.

### 3.1. To What Extent Do Obesity Measurements Agree?

First, we investigated whether two measurements of obesity risks, BMI and WHR, agreed with each other by running Pearson’s Correlation Tests between them, thus splitting them by sex owing to the different body compositions. 

The results suggested that BMI predicts WHR for both male and female participants, with female participants having stronger relationships between BMI and WHR. The statistical tests showed significant correlations between BMI and WHR for both male (F [1, 47] = 7.5, Adj. R2 = 0.12, *p* < 0.05) and female participants (F [1, 46] = 16.0, Adj. R2 = 0.24, *p* < 0.05). However, it should be noted that the adjusted R-squares of the relationships were low, hinting that more factors may contribute to WHR beyond BMI. For example, while both variables measure adiposity, BMI measures overall adiposity, and WHR measures central adiposity. This slightly different focus of measurement might partially explain the lower adjusted R-square of the relationships. 

### 3.2. Doses, Demographics, and WHR

**Figure 3:**
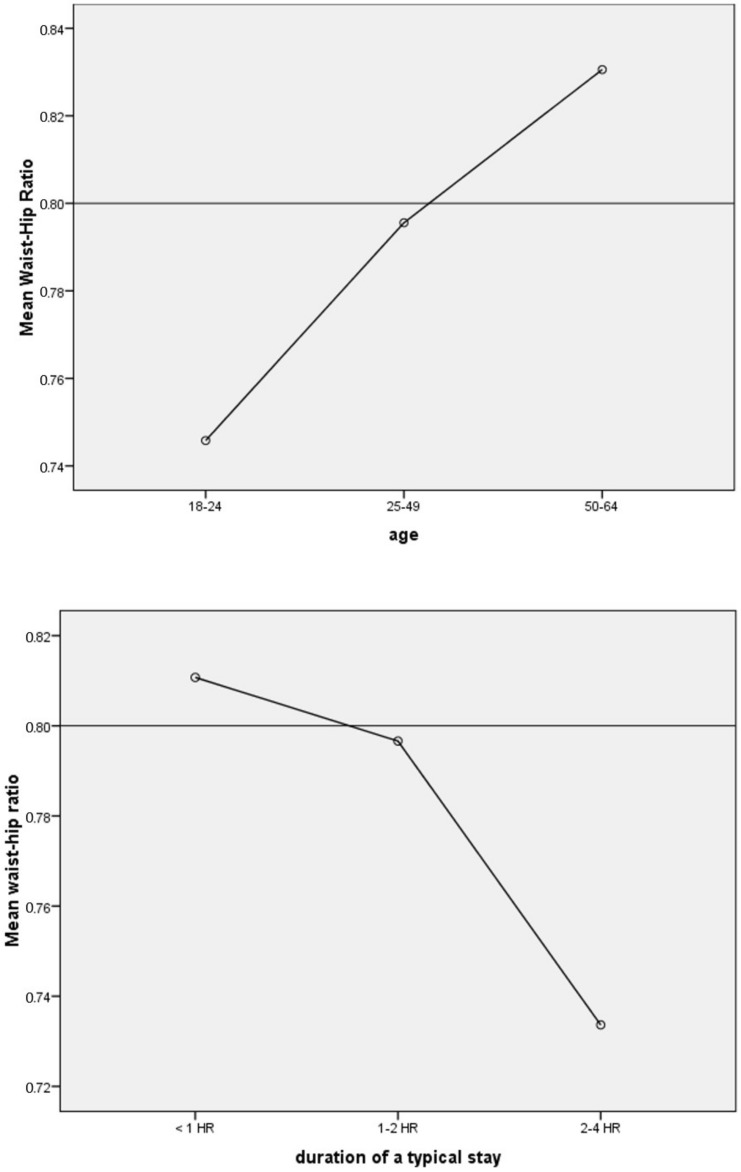
Charts displaying the relationship between age and duration and WHR. The line at 0.8 represented a low-risk WHR, as recommended by WHO [10].

We investigated whether the distance, duration, and frequency correlated with a lower WHR. Based on the existing theories, we expected these factors to contribute to healthier body compositions for both sexes. We found that in this group, the age and duration may predict differences in WHR among women. ANOVA and Welch’s ANOVA suggested significant different WHR values between age and duration groups. However, we did not find other significant relationships (Table 3). We then investigated the nature of the relationships. The results showed that the greater the age, the higher the WHR, but the longer the duration of a typical visit, the lower the WHR (Figure 3).

Because the assumption of equal variances was violated, we could not conduct the two-way ANOVA to investigate the statistical interactions between age and duration and WHR. However, it is likely that there are some interactions between age and duration based on the existing graph results.

### 3.3. Doses, Demographics, and Odds of High-Risk WHR

Next, we tested whether aspects of doses and demographics may predict the odds of having high-risk WHR for both men (0.9) and women (0.8). We found that duration and sex together predicted odds of high-risk WHR among participants. Logistic regression with distance, duration, age, and sex were conducted and revealed that except for distance, other factors were significant predictors of high-risk WHR (Chi-square [4] = 24.9, *p* < 0.001). The model explained 30.7% of the variances (Nagelkerke-R2 = 0.307). The variable frequency was removed because the sample size in its smallest group did not meet the test’s assumption. 

According to the analyses, the odds of high-risk WHR was significantly predicted by gender at the odds ratio of 0.35 for women over men. This suggested that men were found to be 2.9 times more likely to have high-risk WHR than women. Participants’ age predicted the odds of high-risk WHR. In terms of doses, spending more time in green spaces was associated with lower odds of high-risk WHR, while distance to green spaces did not predict the odds. Table 4 presents the statistics of each variable.

## 4. Discussion

### 4.1. Key Findings

The results of this study suggest that first, among female participants, age is associated with higher WHR, and duration in urban nature is associated with lower WHR. This means longer regular visits in nature may predict lower obesity risks. Second, for Asian women whose higher risks WHR is identified as 0.80, a duration of 1–2 h in urban nature is associated with lower WHR (0.79). This suggests that a longer duration in urban nature may sufficiently lower the health risks. Third, sex, age, and duration predicted the odds of having higher obesity risks. Women are less likely to have high-risk WHR compared to men. An older age range predicted higher odds of high-risk WHR, and longer duration in nature predicted lower odds of high-risk WHR. 

### 4.2. Contribution to the Field

This study is congruent with previous studies in that a higher dose of nature is associated with better health outcomes, including reduced obesity risks. Spending 1–2 h in urban nature is also associated with other mental health benefits in some studies [39,41]. However, the duration suggested in this study was a bit higher than the recommended time of physical activities per day, which is 30 min [65]. Additionally, sex and age also tended to predict obesity risks [43]. However, in this study, male participants were reported to have higher obesity risks than women. This is incongruent with most predictions that women are more likely to store fat than men [43,47,49]. However, it is important to note that the measurement of WHR may highlight the body composition in that men are more likely to store fat around their waists, rather than their hips [50]. In terms of the dose–response curve, which may contribute to the growing interest in nature and human relationships [37,38,74], the relationship between the duration of exposure to nature and lower WHR exhibited a linear curve. However, finer categories may be needed to clearly determine the dose–response pattern. 

To the best of the authors’ knowledge, this study is the first to analyze the relationship between the duration of exposure to nature and obesity risks in Thailand and Southeast Asia. It is also the first to examine the link between the dose of nature and WHR, which suitably measures obesity among the Asian population. The study also provided supportive evidence to reveal the positive relationship between urban nature and the physical and mental health of human beings. The growing rate of obesity rate both globally and in Thailand makes the study timely, and the increasing need for other ecosystem services associated with urban nature in Chiang Mai and Thailand, as well as across the world, speaks to the importance of this study.

### 4.3. Recommendations for Practitioners

Overall, the findings support other evidence that urban nature is more than an amenity. Rather, it is an important infrastructure for the urban environment. Thus, designers, planners, and decision makers should provide equally accessible urban nature to urban citizens for recommended WHR and to benefit their overall health and well-being.

### 4.4. Limitations and Future Studies

First, this study used participants who were already visiting the parks, implying that they were participating in physical activities and were in contact with nature. This meant that we used convenience sampling and this might limit the generalizability of the results towards participants outside the geographical region and with different sociodemographic factors. Future studies can address this issue by using other samples. 

Second, the experiment was held only at three places in one city. The diversity and richness of data can be further expanded by comparing more than one place within its subgroups. Third, the study was conducted in a cross-sectional manner, making a record of the changes in participants’ physical health over time impractical. Furthermore, there might be limitations towards causal inference of the results. Longitudinal studies may support the relationship between the duration of nature and obesity. Fourth, the measurements were self-reported. This could lead to measurement errors and biases. Fifth, we require a greater understanding of the quality of green spaces that can induce people to spend around 1–2 h. Thus, designers, planners, and decision makers can incorporate these design elements into urban nature. Sixth, the sociodemographic factors of the participants, such as household income and educational attainment, may limit the interpretability of the findings. Future research may address these factors as the SDoH towards obesity-related issues. 

Lastly, other ecosystem services, such as mitigation, provision, and regulation of natural resources can be considered as co-benefits of green spaces and should be investigated concurrently with the cultural ecosystem services.

## 5. Conclusions

In this study, we conducted a survey among visitors of three urban nature locations in Chiang Mai, Thailand. We asked for their doses of nature, which included distances from green spaces, frequencies of visits, and typical visiting durations, along with their obesity measurements and demographic information. We calculated the risks of obesity from their doses of nature and demographic factors and found that duration of nature predicted lower obesity measurements among women. Additionally, and among all participants, age, gender, and duration predicted lower odds of obesity. Therefore, designers, planners, and decision makers should use the results of this study along with the growing body of evidence to make urban and peri-urban green spaces more accessible to citizens. Finally, future studies should improve upon the generalizability of the study and aim to provide people with equal access to the ecosystem services of urban nature.

## Figures and Tables

**Table 1:** The demographics of the participants across the study sites.

		Rai Mae Hia (*n* = 41)	Nong Buak Had Park (*n* = 34)	Three Kings Monument (*n* = 36)	Total (*n* = 111)	Percent
Age	18–24	13	6	17	36	32.43
	25–49	19	14	13	46	41.44
	50–64	9	14	6	29	26.13
Sex	Male	21	17	19	57	51.35
	Female	20	17	17	54	48.65
Occupation	Physical job	1	3	2	6	5.40
	Desk job	15	13	9	37	33.33
	Homemaking	5	4	2	11	9.90
	Unemployed	2	3	2	7	6.31
	Student	11	3	11	25	22.52
	Other	7	8	10	25	22.52
Living Social Condition	With others	30	27	33	90	81.08
	Alone	10	7	3	21	18.92
Race	Central Thai	11	12	11	34	30.63
	Northern Thai	25	18	17	60	54.05
	Chinese Thai	3	0	4	7	6.31
	Muslim Thai	1	1	1	3	2.70
	Others	1	3	3	7	6.31

**Table 2:** The obesity indices of the participants, separated by gender.

Gender		Height (m)	Weight (kg)	BMI (kg/m^2^)	Waist (in)	Hip (in)	WHR
Male	mean	1.70	71.02	24.74	32.88	36.77	0.90
	S.D.	0.06	22.86	8.61	4.28	3.99	0.07
	maximum	1.82	180.00	69.51	43.00	45.00	1.09
	minimum	1.55	40.00	13.21	21.00	28.00	0.72
Female	mean	1.59	56.30	22.30	29.47	37.23	0.79
	S.D.	0.07	9.72	3.51	4.03	3.73	0.10
	maximum	1.74	97.00	32.04	43.50	48.00	1.21
	minimum	1.45	37.00	15.60	23.00	29.00	0.64
Total	mean	1.65	63.93	23.56	31.22	36.99	0.85
	S.D.	0.08	19.19	6.74	4.48	3.85	0.10
	maximum	1.82	180.00	69.51	43.50	48.00	1.21
	minimum	1.45	37.00	13.21	21.00	28.00	0.64

Note: BMI, body mass index; WHR, waist-hip ratio. We also analyzed the frequencies of participants’ answers from each study site.

**Table 3:** ANOVA and Welch’s ANOVA for the relationships between age, distance, duration, and frequency and WHR.

Independent Variable	Sex	Degree of Freedom	F Value	Assumption of Equal Variances	*p*-Value (or Welch’s *p*-Value)
Age	Men	(2,47)	2.9	0.1	0.07
	**Women**	**(2,44)**	**4.4**	**0.8**	**0.02** *
Occupation	Men	(5,42)	1.1	0.6	0.70
	Women	(5,44)	3.4	1.0	0.48
Living social condition ****	Men	(1,48)	−0.4 ****	0.8	0.67
	Women	(1,46)	−1.4 ****	0.6	0.82
Race	Men	(3,46)	0.39	0.5	0.72
	Women	(4,43)	1.37	4.2	0.05
Distance	Men	(3,46)	0.4	0.4	0.75
	Women	(3,43)	1.5	0.9	0.23
Duration	Men	(2,47)	1.9	0.1	0.16
	**Women**	**(2,44)**	**3.8**	**0.02** **	**0.003** *
Frequency	Men	(4,45)	0.4	- ***	- ***
	Women	(4,42)	1.0	0.5	0.25

* significant relationship at *p* < 0.05. ** Welch’s ANOVA was conducted instead of typical ANOVA. *** the test could not be conducted because one group has fewer than two cases. **** due to the variable having less than 3 categories, independent *t*-test and t-value were used instead of ANOVA and F-value.

**Table 4:** Binary Logistic regressions results between doses, demographics, and high-risk WHR.

Variable	Coefficient	Standard Error	Wald’s Statistics	Degree of Freedom	*p*-Value	OR [95% CI]
**Gender (M)**	**−1.04**	**0.49**	**4.69**	**1**	**0.04** *	**0.35 [0.13, 0.90]**
**Age**	**1.07**	**0.35**	**8.04**	**1**	**0.003** *	**2.91 [1.35, 5.26]**
Occupation	−0.01	0.14	0.01	1	0.93	1.0 [0.75, 1.29]
Living Social condition	0.05	0.33	0.03	1	0.87	1.1 [0.56, 2,00]
Race	0.14	0.23	0.39	1	0.53	1.2 [0.74, 1.81]
Distance	0.175	0.29	0.38	1	0.54	1.22 [0.68, 2.08]
**Duration**	**−0.78**	**0.39**	**3.90**	**1**	**0.046** *	**0.45 [0.21, 1.99]**
Constant	−0.59	1.64	0.00	1	0.97	0.94

* significant difference at *p* < 0.05.

## Data Availability

The data presented in this study are available on request from the corresponding author.
